# Socioeconomic and Employment Status of Patients with Rheumatoid Arthritis in Korea

**DOI:** 10.4178/epih/e2012003

**Published:** 2012-05-07

**Authors:** Jeong-Mi Kwon, Jinnie Rhee, Hyemin Ku, Eui-Kyung Lee

**Affiliations:** Graduate School of Clinical Pharmacy, Sookmyung Women's University, Seoul, Korea.

**Keywords:** Rheumatoid arthritis, Employment, Prevalence, Korea National Health and Nutrition Examination Survey

## Abstract

**OBJECTIVES:**

This study investigates the prevalence of rheumatoid arthritis (RA) by gender and socio-economic characteristics. It also explores the differences in the employment status between RA patients and the general population without RA in Korea.

**METHODS:**

We analyzed data from the Fourth Korea National Health and Nutrition Examination Survey (KNHANES IV) conducted from 2007 to 2009. Prevalence rates were estimated for female and male patients with RA in terms of age, residence, education, income level, and occupation type. The female respondents aged 45 to 64 were divided into the RA population and the non-RA population in order to compare the employment status between the two groups.

**RESULTS:**

The annual physician-diagnosed RA prevalence rate was 1.45%. The prevalence rate was 2.27% for women and 0.62% for men. Individuals with RA had a significantly lower employment rate than individuals without RA (41.7 vs. 68.1%). The main reason for non-employment among RA patients was health-related problems (47.1%). There was statistically significant difference in employment type among the two groups. The experience rates for sick leave and sick-in-bed due to RA were 1.7 and 3.9%, respectively.

**CONCLUSION:**

Middle- and old-aged women accounted for the majority of the Korean RA population, which had a significant lower employment rate compared to the population without RA for both sexes. RA resulted in considerable productivity loss in Korea.

## INTRODUCTION

Rheumatoid arthritis (RA) is a chronic, generally progressive autoimmune disease. The Korean annual self-reported prevalence rate was found to be 2.1% in a previous study [1]. Although the cause of RA remains unclear, RA is known to be associated with a combination of genetic susceptibility and environmental factors, and the difficulty of complete recovery has resulted in the chronic progression of RA [2]. In addition to medical advances in treating RA over the past 10 years, such as novel diagnostic methods and the identification of genetic markers, there have also been improvements in drug therapies such as biologics [3,4].

The chronic progression of RA brings about functional disability and joint degeneration along with pain in the joints, causing limitations in everyday routines, inhibiting the working lives and thus reducing the quality of life for patients [5,6]. Additionally, productivity loss from work disability, absenteeism, and reduction in working hours due to RA may become social problems. RA-induced productivity loss reportedly accounts for more than 50% of the total expenses related to RA treatment, where work disability is found to be the main cause of productivity loss [5,7-11]. Despite the differences in the definition of work disability, research subjects, and research methodologies, various studies have found that the work disability rate of RA patients was high [6,12,13]. As such research is insufficient in Korea, it is necessary to conduct comparative analysis on the employment status of Korean RA patients and that of the general population.

This study investigates the prevalence of RA by gender and socio-economic characteristics. Furthermore, the employment status of RA patients-including employment rate, type of occupation and employment, working hours, and experience of sick leave-are compared with that of the RA-free general population.

## METHODS

### Data source and study population

The Korea National Health and Nutrition Examination Survey (KNHANES) is conducted by the Korea Centers for Disease Control and Prevention. The purpose of KNHANES is to provide representative and reliable statistical data on the health level, health-related attitudes and behavior, and dietary and nutrition status of Koreans. Four surveys have been completed thus far: I (1998), II (2001), III (2005), and IV (2007-2009). Starting with the latest survey, KNHANES IV introduced rolling sampling to ensure the timely report of national statistics. In this study, KNHANES IV-1 [14], KNHANES IV-2 [15], and KNHANES IV-3 [16] datasets were combined and analyzed. The KNHANES IV used a complex sample design based on three-stage sampling (local district (i.e. dong/eup/myeon in Korean) → enumeration district → household). Koreans aged 1 year or older in the sampled households were the subjects of the KNHANES IV.

The KNHANES IV is composed of a health survey (health interview and health consciousness behavior), an examination survey, and a nutrition examination survey. Among them, this study used raw data from a health interview survey. The survey of morbidity in the health interview survey included four questions: "Have you ever been sick?", "Have you been sick for more than three months in the recent year?", "Are you currently sick?", and "Were you diagnosed by a physician?" Through these questions, the annual physician-diagnosed RA prevalence rate among adults aged 19 or more was calculated with the following two conditions: (1) the response that the individual was either sick for over three months in the recent year or currently sick, and (2) the finding that they were simultaneously diagnosed by a physician.

### Employment status and productivity loss

The survey respondents aged 45 to 64 were selected and divided into the population with RA and the population without RA in order to compare the employment rate between the two groups. The analysis was limited to those aged 45 to 64 because prevalence rate for those 19 to 44 was very low, and employment status and sickness were known to be related with age.

As for a respondent who worked for more than one hour in the recent week for income, worked over 18 hours as an unpaid family worker, or was employed but took a temporary leave of absence, he/she was categorized as employed [14-16]. An unpaid family worker refers to a person who worked more than 1/3 of typical working hours without normal wages in a private business operated by family members of kinship.

Analyses on the below items except employment rate were made only on the female subgroup aged 45 to 64, as the number of male respondents aged 45 to 64 classified as the RA patients in the KNHANES IV remained minimal given the nature of RA. A comparative analysis was conducted on reason for unemployment, occupation type, employment type, and average weekly working hours according to whether or not they had RA. The respondents were classified into 6 occupation types (i.e. manager/professional level, office workers, service workers/sellers, agriculture/fishery, technicians/mechanics/assemblers, and simple labor).

Workers were surveyed for their experiences of receiving sick leave in the recent month. For both workers and non-workers, sick-in-bed experiences for which the respondent had to stay in bed almost all day due to sickness or injury in the recent month were analyzed. "Almost all day" refers to staying in bed for more than half a day, such as the morning or afternoon, and having to "stay in bed" refers to a case in which one had to remain lying down in bed, the floor, or on a sofa or when one spent the day lying down watching TV because he/she was sick. In particular, the RA population was surveyed for their experiences of being sick in bed or receiving sick leave in the recent month due to RA.

### Statistical analysis

KNHANES was based on a complex sample design. Therefore, all statistical analyses were performed using the survey procedure of SAS/STAT version 9.1 (SAS Inc., Cary, NC, USA) specifically designed to analyze such sample survey results. In the survey procedure, information pertaining to complex sample designs such as stratification, clustering, and unequal weighting is combined to estimate the parameters.

The annual physician-diagnosed RA prevalence rate was calculated for the entire adult population and compared between men and women. Logistic analyses were conducted to test the statistical significance between the groups (residence, education, household income, individual income, and occupation) by controlling for sex and age. In order to ascertain if there are statistically significant differences in various factors between the RA and non-RA populations, a t-test was utilized for the continuous variables; the Rao-Scott chi-square test (design-adjusted chi-square test) was used for the categorical variables. For all statistical analyses, the results were considered statistically significant when p<0.05.

## RESULTS

### Prevalence rate of RA

Combining the health survey data of KNHANES IV-1 [14], KNHANES IV-2 [15], and KNHANES IV-3 [16], the total adult participants above the age of 19 included in this study was 17,311 (2,983 in KNHANES IV-1, 6,817 in KNHANES IV-2, and 7,511 in KNHANES IV-3). The annual physician-diagnosed RA prevalence rate was estimated to be 1.45% (95% confidence interval [CI], 1.27 to 1.64). The prevalence rate for women was 2.27% (95% CI, 1.96 to 2.57), 3.7 times greater than the prevalence rate of 0.62% (95% CI, 0.43 to 0.82) for men.

The prevalence rate increased with age for both sexes, and with a lower education level. The prevalence rate for adults in their 50s and above was over 2%, whereas that for adults under 40s was less than 1%. Also, those with high school/bachelor/graduate education showed a 1% or lower prevalence rate, while the figure for elementary school graduates or those with lower education was 4.36%. The prevalence rate was found to be higher in rural areas than urban areas, increasing with lower household income levels. Among occupation types, workers in the agriculture and fishery industries were found to have high prevalence rates for both sexes. These trends were more pronounced among women. However no distinct trend was found between the prevalence rate and the individual income level (Table 1).

According to the logistic analyses, the odds of RA prevalence for female respondents were 3.15 (95% CI, 2.24 to 4.45) times higher than those of their male counterparts and were statistically significant. The prevalence rate increased with age (odds ratio [OR], 1.06; 95% CI, 1.05 to 1.07), and decreased with higher education (high school: OR, 0.41, bachelor/graduate: OR, 0.18). These differences were statistically significant when controlling for sex and age. However, there was no statistically significant difference of prevalence rate by residence, household income level, individual income level, and occupation after controlling for sex and age (Table 2).

### Employment status of RA patients

The population aged 45 to 64 was divided into the RA population and the non-RA population. The number of respondents included in this analysis of employment status was 133 in the RA population and 5,774 in the non-RA population. The number of female respondents among them stood at 114 and 3,221, respectively. When this was used to estimate the total population with (or without) RA in Korea, the total RA population and total non-RA population were found to include 236,265 and 12,061,610 adults aged 45 to 64 years, respectively. The employment rate of the total population without RA was 68.1% (95% CI, 66.3 to 69.8), while it was 41.7% (95% CI, 31.8 to 51.5) for the total population with RA, showing a statistically significant difference. The difference in employment rate between the RA and non-RA populations was observed in both sexes: 83.4% (95% Cl, 81.6 to 85.2) and 63.2% (95% Cl, 38.8 to 87.6) for men and 52.3% (95% Cl, 50.0 to 54.7) and 35.3% (95% Cl, 25.8 to 44.8) for women. The employment rate of the RA population was statistically significantly lower than that of the non-RA population for both male and female respondents (Table 3).

As indicated earlier in research methodology, the number of male respondents aged 45 to 64 classified as the RA population in the KNHANES IV was a meager 19 out of 133 in total. Therefore, all the analyses described below are only about female respondents in the same age group.

An analysis of the causes of unemployment among individuals in between jobs showed that the main reason for unemployment was "health-related problems" for both populations, though the proportion of such responses was higher in the RA population (Table 4).

Among the occupation types for employed individuals, the two groups showed similar distribution by occupation type, except for the proportion of the response "agriculture/fishery" being higher for the RA population (Table 5). However, there was a statistically significant difference between employment types for the non-RA population and the RA population. In the population without RA, the weight of wageworkers was the highest at 55.6%. In the population with RA, however, the proportion of unpaid family workers was relatively high at 29.7% (Table 5).

The analysis of sick leave experiences due to disease or injury in the recent month showed that the proportion of those with sick leave experience was 6.7% in the non-RA population and 9.7% in the RA population, which did not constitute a statistically significant difference. Similarly, the sick-in-bed experience was 9.4% in the non-RA population and 10.0% in the RA population. In the recent month the sick leave experience due to RA was 1.7%, and the sick-in-bed experience due to RA was 3.9% (Table 6).

## DISCUSSION

This study investigated the prevalence characteristics of RA by gender in the adult population aged 19 or over and performed comparative analysis of employment characteristics between the population with RA and the population without RA within the population aged 45 to 64.

The RA prevalence rate of the Korean population was found to be approximately 1.45%, and the prevalence rate of women was 3.7 times greater than that of men, which is in agreement with previous research [1,5]. The gap in prevalence by socio-economic characteristics would be mostly attributable to the proportion and age of female respondents in each group. This is because there was no statistical significant difference in the prevalence rate by socioeconomic status after controlling for sex and age, except the prevalence rate by education level.

The employment rate of the RA population was found to be significantly lower than that of non-RA population for both sexes. More than half the RA population aged 45 to 64 was unemployed, and a majority of the unemployed RA population could not be employed due to health reasons. It is predicted that work disability due to RA will likely increase as the rate of economic activity participation by women continues to increase in the future [6,11]. Compared to the non-RA population, RA population showed a high tendency to be unpaid family workers rather than to be wageworkers, self-employed, or employers participating in economic activities. This implies a low income level for the RA population. For the wageworkers, however, weekly working hours made no difference between the RA and non-RA populations.

There were no statistically significant differences in the proportion of those with sick leave experience due to disease or injury in the recent month, but the figure was found to be high for RA patients. In this study, the proportion of those having taken sick leaves due to RA was 1.6% for RA patients, which is lower than what is observed elsewhere around the world. The proportion of individuals with sick leave experience in the recent three months was found to be 33% in Germany [17]; those with such experience in the recent two weeks accounted for 16% in the Netherlands [18]. A similar finding to us was also reported in a study involving the American population [19]. The low proportion of those with sick leave experience can be understood in the context of Korean workplace culture where negative views prevail on those taking sick leave as well as the psychological pressure facing workers who are concerned about the potential disadvantages of taking sick leaves [20].

This study has several limitations. Due to the nature of the cross-sectional survey used in this case, the recall bias cannot be eliminated. The sample design of KNHANES was intended to represent the Korean population; the fact that there were only 133 RA patients aged 45 to 64, however, may be regarded as a limitation. In particular, only 19 out of the 133 respondents were men. For this reason, the standard error was large in the detailed analysis. In addition, special attention is required when interpreting the results: though both the RA and non-RA populations were confined to women aged 45 to 64 when analyzing the association between RA and employment status, the two groups may feature different socio-demographic characteristics that this study could not take into account.

Despite these limitations, the significance of this study lies in its presentation of the latest socio-economic prevalence characteristics of RA and the employment conditions of RA patients using the health interview survey data of KNHANES IV. Because KNHANES is a periodically conducted nationwide survey, the present study would be meaningful as preliminary research in case similar research is pursued employing KNHANES data. In addition, this study would have greater significance as preliminary data when considering societal productivity loss due to RA.

The results of this study show that elderly women account for the majority of the Korean RA patient population, and that the prevalence rate increases with age for both sexes and with a lower education level. Furthermore, the Korean RA population has a much lower employment rate compared to the population without RA for both sexes. For this reason, RA is expected to result in considerable productivity loss in Korea as well.

## Figures and Tables

**Table 1:**
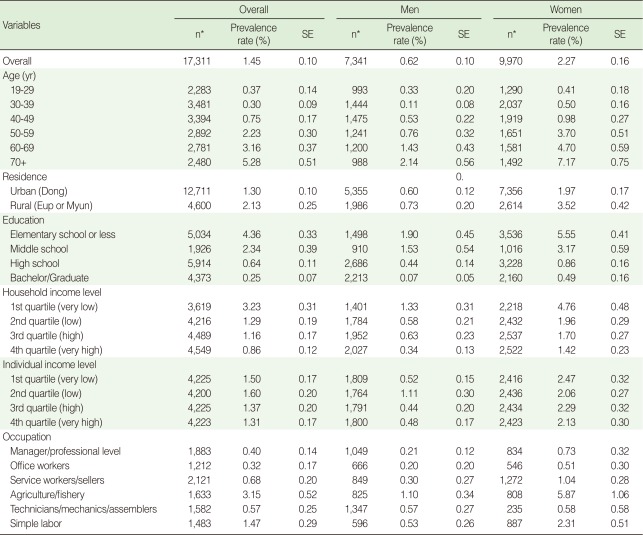
Prevalence rate of rheumatoid arthritis according to age, sex and socio-economic characteristics

SE, standard error. ^*^KNHANES IV participants.

**Table 2:**
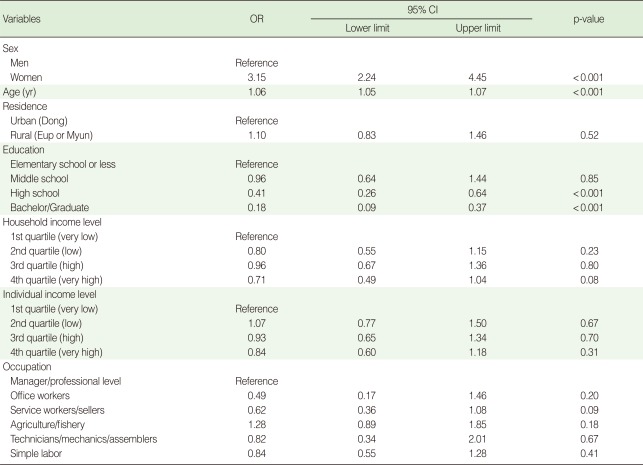
Sex- and age-adjusted odds ratios of rheumatoid arthritis with socio-economic characteristics by logistic regression analysis

OR, odds ratio; CI, confidence interval.

**Table 3:**
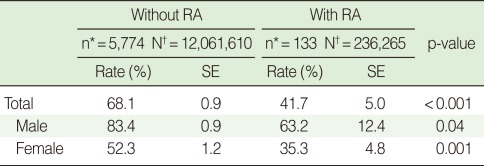
Employment rates of individuals with and without rheumatoid arthritis (RA) aged 45 to 64

SE, standard error. ^*^KNHANES IV participants; ^†^Estimated number of the Korean population aged 45-64.

**Table 4:**
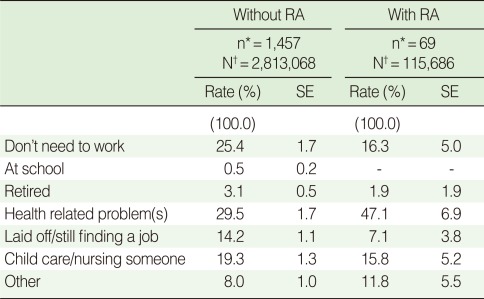
Reasons for unemployment of unemployed female individuals with and without rheumatoid arthritis (RA) aged 45 to 64

SE, standard error ^*^KNHANES IV participants; ^†^Estimated number of the Korean female population aged 45-64.

**Table 5:**
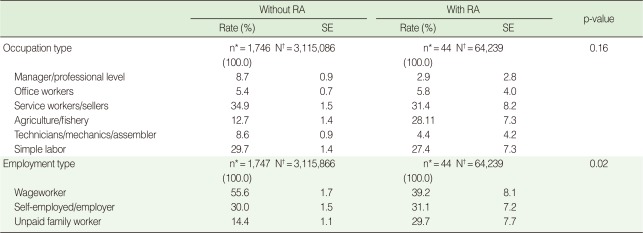
Types of occupation and employment of employed female individuals with and without rheumatoid arthritis (RA) aged 45 to 64

SE, standard error. ^*^KNHANES IV participants; ^†^Estimated number of the Korean female population aged 45-64.

**Table 6:**
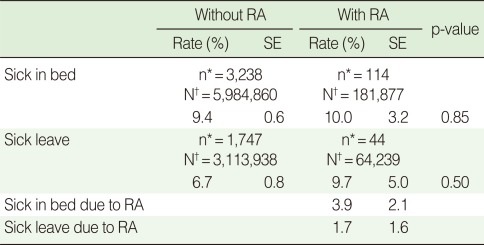
Experience rates for sick in bed and sick leave of employed female individuals with and without rheumatoid arthritis (RA) aged 45 to 64

SE, standard error. ^*^KNHANES IV participants; ^†^Estimated number of the Korean female population aged 45-64.
